# Dietary diversity and food security status among heart failure patients in the north of Iran

**DOI:** 10.1186/s40795-021-00438-y

**Published:** 2021-07-09

**Authors:** Marjan Mahdavi-Roshan, Azin Vakilpour, Seyed Mehdi Mousavi, Asieh Ashouri

**Affiliations:** 1 Cardiovascular Diseases Research Center, Department of Cardiology, Heshmat Hospital, School of Medicine, Guilan University of Medical Sciences, Rasht, Guilan Iran; 2 Department of Nutrition, School of Medicine, Guilan University of Medical Sciences, Rasht, Iran; 3 Research Center of Health and Environment, School of Health, Guilan University of Medical Sciences, Rasht, Iran

**Keywords:** Dietary diversity score, Food frequency questionnaire, Food insecurity, Heart failure, Household food insecurity access scale

## Abstract

**Background:**

Dietary diversity score (DDS) is an indicator for assessing nutritional adequacy. Food security is another important measure in nutrition field which can be associated with several cardiovascular risk factors. Considering the importance of nutrition in heart failure (HF) patients, this study was designed to evaluate the DDS and food security of patients with HF.

**Methods:**

A total of 200 HF patients were enrolled. DDS was evaluated using valid and reliable food frequency questionnaire and was calculated by scoring food intakes as 5 main groups. Household food insecurity access scale was applied to assess food security status. Data were analyzed using descriptive statistics, Chi-square and Kruskal-Wallis tests and multiple logistic regression models.

**Results:**

The mean age of patients was 65 (standard deviation: 12) years and 59% of patients were male. Median of DDS was 1.96 (range: 0.29 to 6.12). Adjusted odds of greater DDS (> = median of 2) was 2.58 times higher for patients without hypertension than for patients with hypertension (95%CI: 1.31–5.08, *P* = 0.006). Also, odds of greater DDS were more in ex-smokers’ patients when compared to non-smokers (adjusted odds ratio (AOR): 2.70, 95%CI: 1.27–5.75, *P* = 0.010), patients with supplement use (AOR: 2.42, 95%CI: 1.16–5.05, *P* = 0.019), patients with lower total cholesterol level (AOR: 1.01, 95%CI: 1.00–1.02, *P* = 0.051), and patients with higher ejection fraction (AOR: 1.03, 95%CI: 1.00–1.05, with borderline.

*P* = 0.073). About 57% of patients had experienced degrees of food insecurity as mild (26%), moderate (16%) and severe (15%). On the other hand, women (AOR: 1.90, 95%CI: 0.90–3.71, with borderline *P* = 0.061) and patients with middle (AOR: 3.48, 95%CI: 1.79–6.76, *P* < 0.001) or high (AOR: 20.32, 95%CI: 2.56–161.19, *P* = 0.004) socio-economic status were more likely to be food secure or mild insecure. Also, no relation between DDS and food security was found (*r* = − 0.08, *P* = 0.262).

**Conclusion:**

This study found that HF patients had a low DDS and more than half of the patients were food insecure to some extents.

## Background

Heart failure (HF) is an advanced health problem around the world which imposes high costs on health care systems [1]. Prevalence of HF is increasing and it is estimated that about 26 million people worldwide are influenced by the disease [2].This is due to increased prevalence of its known risk factors such as: diabetes, hypertension, hyperlipidemia, ischemic heart disease, obesity and overweight [1]. A vast body of literature have shown the relationship between dietary factors and cardiovascular diseases prevention [3–6]. Cardiovascular diseases (CVDs) are one of the most significant medical challenges and are responsible for more than one third of deaths in Iran [7]. HF is caused by many cardiovascular diseases, the most important of which is myocardial infarction [8]. The prevalence of HF is reported between 0.4 to 4.3% in general population and has been estimated about 8% in Iranian population which is higher than other countries in this region [7, 9]. Owing to the importance of HF and the increasing rate of complications resulting in mortality, the need to develop healthcare strategies and to manage patients’ quality of life is growing [1, 8]. Frequency of nutritional inadequacy in patients with chronic HF is prominent and it is estimated that about 75–90% of patients with advanced HF suffer from malnutrition [2]. Energy protein malnutrition is often common in patients with HF because of increased energy needs for heart and lung function as well as decreased desire to eat food due to weakness and early satiety, besides, Risk of electrolyte imbalance and nutrients deficiency as a result of drug use such as diuretics in patients with HF indicate the necessity of paying attention to diet and adequate nutrition [10, 11].

Dietary diversity score (DDS) is a valuable evaluation index and useful indicator for assessing nutritional adequacy. Dietary diversity constitutes a qualitative measurement of food consumption that accounts for household access to a variety of foods. It is also an indicator of the nutritional sufficiency of the diet of individuals [12, 13]. Taking into consideration the impact of dietary diversity on the supply of nutrients and vitamins, it is one of the most important components of a healthy diet [14, 15]. Having more variety in the daily diet is in association with more macro and micronutrients intake. Previous surveys have indicated that DDS may be relevant to the prevalence of some non-communicable chronic disease such as CVDs, malignancies and metabolic syndrome [16, 17]. The greater the dietary diversity of a diet is, the more protection it can provide against many chronic diseases [18–20].

Another important indicator in the field of nutrition is food security that means a situation in which all people have physical and economical access to adequate, healthy and nutritious food all the times and available foods provide the needs of a nutritious diet in harmony with people’s interests to live an active, healthy life [21]. There have been two main aspects in all definitions of food security: 1-physical access to food which means availability of food in the environment 2 – Economic access in the form of having sufficient purchasing power to obtain food from the market or gain access to raw materials for production. Lack of access through acceptable ways or unreliable access to healthy foods is defined as food insecurity [22, 23]. Studies have shown that food insecurity can be associated with many health problems including CVDs risk factors such as hypertension, hyperlipidemia, diabetes, over weight and obesity [24, 25]. According to the food and agriculture organization in 2017, about 10% of the world’s population are experiencing severe food insecurity [26].

Considering the importance of nutrition in patients suffering from HF disease, this study was designed to assess DDS and food security and some related factors among HF patients.

## Methods

### Participants

This cross-sectional study was conducted on heart failure patients referring to Dr. Heshmat hospital, the only specialized center of cardiovascular diseases in Guilan province, Iran between December 2018 and November 2019.

Male and female in the age range of 20–70 years with HF with reduced ejection fraction (LVEF≤40%) who has not been on any special diet for one month before the study were included. The diagnosis of HF was confirmed by a cardiologist. Patients were included in the study if they had risk factors such as hypertension, diabetes, hyperlipidemia and obesity. On the contrary, incomplete questionnaires and individuals with no inclination to participate at any time of the study were excluded. Until the completion of the sample size, all consecutive patients who met the inclusion criteria were enrolled in the study.

### Data collection

After explaining the purpose of the study and obtaining informed consent, data was gathered. Demographic characteristics (such as sex, age, marital status, socioeconomic status based on household monthly income, household size, number of children, history of smoking, educational level, occupation) were collected by interview. Taking at least one of the Iron, Zinc, vitamin D, vitamin B groups, vitamin C and E supplements was recorded, as well. Anthropometric profile (such as height, weight, body mass index (BMI), waist circumference and loss of weight during last 6 months) were measured by trained healthcare providers and clinical information and medical history were gathered by reviewing the medical records of patients.

### Dietary diversity assessment

Dietary diversity is a qualitative measure of food consumption that considers household access to various foods and is also an indicator of the adequacy of nutrients in a person’s diet [12, 13]. In order to evaluate dietary diversity, a 168 items semi-quantitative food frequency questionnaire (FFQ) was used [27]. Previous studies in Iran have evaluated its validity and reliability [13, 28]. Kant et.al method was used to score dietary diversity [29]. According to the food pyramid by the U.S department of agriculture, five main food groups including: bread and cereals, vegetables, fruits, meats and dairy products, were considered in the calculation of DDS. The main food groups then were divided into 23 subgroups, which showed the food diversity within each of the pyramid food groups. Subjects were questioned how often they consumed the given serving of each food item during the past year on a daily, weekly or monthly basis. If a person consumed at least half a portion of a food subgroup in a day, he/she was accounted as a consumer of that subgroup. Maximum food diversity score assigned to each of the five groups was 2. As such, a total score of 0 (min) to 10 (max) could be calculated from the sum of these numbers. The minimum score of dietary diversity is zero and the maximum is 10. The closer the dietary diversity score to 10, the more appropriate the dietary diversity is in the subjects [3, 30].

### Food security assessment

Food security assessment (food access) was performed using household food insecurity access scale (HFIAS) [31] which has been translated into Persian and validated using exploratory factor analysis in a previous study [32]. It includes a set of 9 questions in order to measure food access component of the household. For each question, five options about the number of times of experiencing the situation (0 = never, 1-rarely once or twice, 2-sometimes (three to ten times), 3-often (more than ten times) during the period of one month (past 30 days) are considered. Obtained data can be used both quantitatively and qualitatively to assess food insecurity in the study population. The lowest score is 0 and the highest is 27. This criterion divides households into four groups in terms of security: safe (0–1 Points), mild insecurity (2–7 Points), moderate (8-14 Points) and severe (15–27 Points). The higher the household score, the greater the food insecurity is experienced [33].

### Statistical analysis

The sample size of this study was determined based on Najibi et.al study [34]. A total of 200 patients were calculated on the basis of estimating about 67% prevalence of food insecurity, with confidence interval of 95% and accuracy of 7%, then adding 15% higher rate to compensate for the possible dropout.

Data were presented as frequency (percent), median (range) or mean (standard deviation (SD)). Chi-square test was used to investigate the relation between food diversity and patients’ qualitative characteristics. Kruskal-Wallis test was used to investigate the relation between food diversity or food security and patients’ quantitative characteristics. Spearman correlation coefficient test was used to investigate the relation between food diversity score and food security. Multivariable logistic regression models with stepwise backward method were used to identify characteristics independently associated with the food diversity or food security. All variables with significant level of 0.1 in the univariate analyses were assessed in the multivariable models. In the multivariable logistic regression model, dependent variables of DDS were grouped based on median and food insecurity were grouped based on complete security or mild insecurity compared to moderate or severe insecurity status. All analyses were performed by SPSS software version 21.

## Results

Patients’ demographic, anthropometric and clinical characteristics are shown in Table 1. Of all 200 patients included in the study, 59% were male. The mean age was 65 years (SD = 12.1). Seventy-five percent of patients were married, 23% were widowed, 1% were divorced and 1% were single. Majority of patients (87%) had educational level lower than high school. Sixty-five % of the subjects were non-smokers and 24% stated that they had a history of smoking but no longer smoked.

**Table 1:** Demographic, anthropometric and clinical characteristics of patients

Characteristic	Total (***n*** = 200)
Sex, no. (%)	
Male	118(59)
Female	82(41)
Age (years), mean (SD, range)	65(12.1, 20–89)
Education, no. (%)	
Illiterate	96(48)
Primary	47(23)
Secondary	30(15)
Diploma	19(10)
Academic	8(4)
Household size, no. (%)	
1	30(15)
2	89(45)
3	49(24)
4 or more	32(16)
Socioeconomic status, no. (%)	
Low	85(42)
Middle	95(48)
High	20(10)
Smoking status, no. (%)	
Non-smoker	130(65)
Former-smoker	48(24)
Current smoker	22(11)
BMI category, no. (%)	
Under weight	2(1)
Normal	66(33)
Overweight	80(40)
Obese	52(26)
Weight loss in the last 6 months (yes), no. (%)	87(44)
Waist circumference (cm), mean (SD)	95 (25)
Supplement use^a^, no. (%)	46(23)
History of disease, no. (%)	
Heart disease	70(35)
Diabetes	99(50)
Hypertension	131(66)
Hyperlipidemia	74(37)
Heart failure duration (years), mean (SD, range)	2.6(3.6, 0.1–21)
FBS (mg/dl), mean (SD)	146(67)
TC (mg/dl), mean (SD)	142(47)
TG (mg/dl), mean (SD)	112(61)
HDL (mg/dl), mean (SD)	39(10)
LDL (mg/dl), mean (SD)	80(39)
Ejection fraction (%), mean (SD)	27(11.7)

*SD* indicated standard deviation, *BMI* body mass index, *FBS* fasting blood sugar, *TC* total cholesterol, *TG* triglyceride, *HDL* high density lipoprotein, *LDL* low density lipoprotein, *BUN* blood urea nitrogen, *NA* sodium, *K* potassium

^a^Supplement use was indicated as taking at least one of the Iron, Zinc, vitamin D, vitamin B groups, vitamin C and E supplementations

Mean of BMI was 27.40 kg/m^2^ (SD: 5.23) and 44% of the patients reported having weight loss during past 6 month. The mean duration of suffering from HF was 2 years (range: < 2 month to 21 years), and about 50% of patients had a history of diabetes, 66% had hypertension, and also 23% reported taking supplements (Iron, Zinc, Vitamin D, vitamin B groups, vitamin C and E) (Table 1).

The median score of DDS was 1.96 (range: 0.29 to 6.12; mean: 2.10, SD: 0.92) and the highest to lowest food intake group based on the medians belonged to fruits, bread and cereals, dairy products, vegetables, and meats, respectively (Table 2).

**Table 2:** Patients dietary diversity score based on Kant (*n =* 200)

DDS of food pyramid groups^a^	Median	Mean	SD	Min.	Max.
**Total score**	1.96	2.10	0.92	0.29	6.12
Bread and cereals	0.57	0.67	0.24	0	1.14
Bean and vegetables	0.29	0.20	0.23	0	1.14
Fruits	0.67	0.44	0.46	0	2.00
Meats	0.00	0.26	0.36	0	2.00
Dairy products	0.40	0.53	0.34	0	2.00

*DDS* indicated dietary diversity score, *SD* standard deviation

^a^The range of score in each food group is 0 to 2 and the range of score in total is 0 to 10

The median score of food security was 2.5 (range: 0 to 26; mean: 5.31, SD = 6.33). About 57% (95%CI: 50 to 64%) of patients had experienced degrees of food insecurity, categorized as mild (26, 95%CI: 20 to 32%), moderate (16, 95%CI: 11 to 22%) and severe (15, 95%CI: 11 to 20%).

Univariable analyses showed that patients with a history of hypertension (*P* = 0.026), patients who did not take supplements (*P* = 0.044) and those with a longer duration of suffering from HF (*p* = 0.009) had significantly less dietary diversity; while former smokers compared to non-smokers had significantly greater diversity in their diet (*P* = 0.006) (Table 3). In the multiple logistic regression model, hypertension, smoking, taking supplement, total cholesterol level and left ventricular ejection fraction level (LVEF) were entered in the model. Patients without hypertension (adjusted odds ratio (AOR): 2.58, 95%CI: 1.31–5.08, *P* = 0.006), ex-smokers compared to non-smokers (AOR: 2.70, 95%CI: 1.27–5.75, *P* = 0.010), patients with supplement use (AOR: 2.42, 95%CI: 1.16–5.05, *P* = 0.019), patients with lower total cholesterol level (AOR: 1.01, 95%CI: 1.00–1.02, *P* = 0.051) and higher LVEF (AOR: 1.03, 95%CI: 1.00–1.05, *P* = 0.073) were more likely to have DDS of 2 or more (Table 4).

**Table 3:** Dietary diversity score and food security status by demographic, anthropometric and clinical characteristics of patients

Characteristic	Dietary diversity score			Food secure status				
	< 2 (***n =*** 100)	> = 2 (***n =*** 100)	P*	Secure (***n*** = 86)	Mild insecure (***n*** = 52)	Moderate insecure (***n*** = 32)	Severe insecure (***n*** = 30)	P*
Sex, no. (%)			0.774**					0.032
Male	58(49)	60(51)		56(47)	22(19)	19(16)	21(18)	
Female	42(51)	40(49)		30(37)	30(37)	13(16)	9(11)	
Age (years), mean (SD)	66 (12.3)	65 (12.0)	0.515	66(11.0)	65(13.7)	62(14.5)	69(8.9)	0.312
Education, no. (%)			0.529					0.026
Illiterate	51(53)	45(47)		33(34)	30(31)	14(15)	19(20)	
Primary	25(53)	22(47)		20(43)	11(23)	6(13)	10(21)	
Secondary	14(47)	16(53)		14(47)	8(27)	8(27)	0(0)	
Diploma	8(42)	11(58)		13(68)	3(16)	3(16)	0(0)	
Academic	2(25)	6(75)		6(75)	0(0)	1(13)	1(13)	
Household size, no. (%)			0.430					0.270
1	13(43)	17(57)		17(57)	3(10)	4(13)	6(20)	
2	46(52)	43(48)		37(42)	25(28)	11(12)	16(18)	
3	28(57)	21(43)		20(41)	15(31)	11(22)	3(6)	
4 or more	13(41)	19(59)		12(38)	9(28)	6(19)	5(16)	
Socioeconomic status, no. (%)			0.303**					0.001
Low	46(54)	39(46)		31(36)	14(16)	19(22)	21(25)	
Middle	47(49)	48(51)		44(46)	30(32)	12(13)	9(9)	
High	7(35)	13(65)		11(55)	8(40)	1(5)	0(0)	
Smoking status, no. (%)***			0.030**					0.439
Non-smoker	72(55)	58(45)		54(42)	39(30)	19(15)	18(14)	
Former-smoker	16(33)	32(67)		20(42)	10(21)	8(17)	10(21)	
Current smoker	12(55)	10(45)		12(55)	3(14)	5(23)	2(9)	
BMI category, no. (%)			0.513					0.794
Under weight	1(50)	1(50)		0(0)	1(50)	1(50)	0(0)	
Normal	29(44)	37(56)		25(38)	19(29)	12(18)	10(15)	
Overweight	45(56)	35(44)		35(44)	19(24)	13(16)	13(16)	
Obese	25(48)	27(52)		26(50)	13(25)	6(12)	7(13)	
Weight loss in the last 6 months (yes), no. (%)	42(48)	45(52)	0.669**	34(39)	29(33)	11(13)	13(15)	0.185
Waist circumference (cm), mean (SD)	97(23)	94(26)	0.101	97(22)	87(15)	97(32)	104(33)	0.048
Supplement use, no. (%)	17(37)	29(63)	0.044**	20(43)	13(28)	7(15)	6(13)	0.961
History of disease, no. (%)								
Heart disease	34(49)	36(51)	0.767**	34(49)	18(26)	12(17)	6(9)	0.280
Diabetes	55(56)	44(44)	0.120**	43(43)	29(29)	15(15)	12(12)	0.573
Hypertension	73(56)	58(44)	0.026**	55(42)	38(29)	23(18)	15(11)	0.159
Hyperlipidemia	36(49)	38(51)	0.770**	31(42)	24(32)	10(14)	9(12)	0.394
Heart failure duration (years), mean (SD)	3.2(4.32)	2.0(2.56)	0.009	2.8(3.82)	2.5(4.03)	2.3(2.98)	2.7(2.77)	0.202
FBS (mg/dl), mean (SD)	146(66)	145(69)	0.566	151(70)	159(80)	127(4)5	128(45)	0.102
TC (mg/dl), mean (SD)	148(57)	136(35)	0.103	143(58)	149(44)	135(29)	135(33)	0.447
TG (mg/dl), mean (SD)	120(76)	103(39)	0.347	113(58)	122(76)	103(65)	102(29)	0.452
HDL (mg/dl), mean (SD)	39(10)	38(10)	0.398	39(10)	38(10)	39(11)	40(10)	0.916
LDL (mg/dl), mean (SD)	84(46)	76(30)	0.338	80(47)	87(33)	74(25)	75(31)	0.331
Ejection fraction (%), mean (SD)	25(11)	28(12)	0.108	26(11)	27(13)	26(11)	29(12)	0.600

*SD* indicated standard deviation, *BMI* body mass index, *FBS* fasting blood sugar, *TC* total cholesterol, *TG* triglyceride, *HDL* high density lipoprotein, *LDL* low density lipoprotein, *BUN* blood urea nitrogen, *NA* sodium, *K* potassium

* *P*-value was reported from Kruskal-Wallis test except otherwise indicated

** *P*-value was reported from Chi-square test

*** Based on the post-hoc paired comparisons, only former-smokers compared to non-smokers had significantly greater diet diversity (*P =* 0.006) and other comparisons was not significant ((*P* > 0.05 for all)

**Table 4:** Odds ratios and 95% confidence intervals of being in dietary diversity scores > =2 or being in food secure or mild insecure status by characteristics of patients independently related to dietary diversity score or food secure status

Characteristic	Dietary diversity score		Characteristic	Food secure status	
	Adjusted OR (95% C) ^**a**^	P		Adjusted OR (95% CI)^**a**^	P
Smoking status		0.036	Sex		0.061
Non-smoker	1.00		Male	1.00	
Former-smoker	2.70 (1.27–5.75)	0.010	Female	1.90 (0.97–3.71)	
Current smoker	1.31 (0.47–3.66)	0.604	Socioeconomic status		< 0.001
Supplement use		0.019	Low	1.00	
No	1.00		Middle	3.48 (1.79–6.76)	< 0.001
Yes	2.42 (1.16–5.05)		High	20.32 (2.56–161.19)	0.004
Hypertension disease		0.006			
Yes	1.00				
No	2.58 (1.31–5.08)				
TC	0.99 (0.98–1.00)	0.051			
Ejection fraction	1.03 (0.99–1.05)	0.073			

^a^To predict dietary diversity scores > = 2 or being in food secure or mild insecure status based on patients’ characteristics, a backward stepwise multiple logistic regression was performed

*TC* indicated total cholesterol, *OR* odds ratio, *CI*, confidence interval

Also, univariable analyses in assessing relation between patients’ characteristics and food security status showed that men were more likely to experience severe food insecurity than women (*P* = 0.032), waist circumference was higher in patients who reported more food insecurity (*P* = 0.048) and patients who had poor economic status were significantly more likely to have food insecure status (*P* = 0.001). Also, patients with higher education were experiencing higher levels of food security (*P* = 0.026) (Table 3). The results of multiple logistic regression model showed that only patients’ gender and socio-economic status were independently related to the food security status.

Based on the data, although the overall prevalence of food insecurity among women was higher than men, women experienced more mild level of food insecurity and odds of being in food secure or mild insecure status was more in women compared to men (AOR: 1.90, 95%CI: 0.90–3.71, *P* = 0.061) and men on the other hand experienced more severe level of food insecurity. Also, Patients with middle (AOR: 3.48, 95%CI: 1.79–6.76, *P* < 0.001) or high (AOR: 20.32, 95%CI: 2.56–161.19, *P* = 0.004) socio-economic status compared to low socio-economic status were more likely to be in complete food security or mild insecurity status (Table 4).

There has not been any statistically significant correlation between food security and dietary diversity (*r* = − 0.080, *P* = 0.262).

## Discussion

Adequate nutrition for people suffering from HF disease is of high importance because of the imbalance of electrolytes and vitamins and lack of micronutrients due to the use of diuretics [10, 11]. According to dietary guidelines, dietary diversity is one of the components of a healthy diet. Eating a variety of diets provides the most protection against chronic diseases [35, 36]. There is very limited information about cardiac patients’ nutritional status in north of Iran [37]. To the best of our knowledge this study is one of the first studies evaluating DDS and food security in HF patients.

This study conducted among patients with HF showed high prevalence of low dietary diversity in subjects (Mean DDS = 2.10 ± 0.92). Regarding the assessment of DDS in relation to CVDs risk factors, mean dietary diversity score (mean DDS = 4.71) obtained in Farhangi et al. study was higher than this study [4]. In the study of Dere KAL et al. who investigated DDS in diabetic and hypertensive patients, average individual DDS was reported to be poor, although mean of obtained DDSs in both groups of patients were higher than present study [5]. Also, mean DDS in Azadbakht et al. research evaluating the association of DDS and CVDs risk factors was found higher than this study [3]. Iran is a multi-ethnic country with a rapid nutrition and health transition which results in significant alterations in nutrition status of the population [38, 39]; Therefore, dietary insufficiency and specific nutrients deficiencies characterize the diet of the people, and overeating and obesity are evident in over a third of the population [40, 41].

In present study the most consumed food groups belonged to fruits and grains, however, Azadbakht et al. study which investigated the association of DDS with metabolic syndrome in tehranian adults reported that dairy products, vegetables and fruits, were most used by the study subjects, respectively [6]. In Dere K et al. research the most consumed food groups belonged to vegetables (onion, tomato and peppers), cereals, fish, oils and fats in both diabetic and hypertensive groups while fruits, other vegetables, nuts and seeds were the least consumed [5]. The differences between studies can be considered as a result of differences in characteristics of study population, various sample size and different questionnaire use. Furthermore, geographical and cultural aspects can highly affect diet and nutrition habits which should not be ignored.

Present survey reported a significant relationship between duration of suffering from HF, hypertension, smoking status and dietary diversity. Subjects with hypertension, longer duration of suffering from HF and patients who were not taking any supplement had less varied diet while those who quit smoking (former smokers) showed greater dietary diversity in comparison to non-smokers. In addition, our results showed that individuals with high cholesterol level and low ejection fraction were more likely to experience low diversity in their diet.

On the basis of Azadbakht et al. study, carried out on tehranian adults, dietary diversity had an inverse association with metabolic syndrome, high blood pressure, high triglyceride level and abnormal glucose levels. In their study, participants with greater DDS reported consuming healthier food groups. Hence, they concluded that greater dietary diversity might be in association with lower possibility of having some metabolic disorders [6]. Another research in Iran showed that people with higher dietary diversity score had lower risk of hypertension [3]. Also, Farhangi et al. found that patients with lower DDS had significantly higher serum triglycerides and systolic blood pressure value [4]. This can possibly be attributed to a healthier lifestyle related to higher DDS such as using more fiber, fruits, vegetables and lower intake of meat and cholesterol [3].

All these findings demonstrate that dietary diversity can be associated with some CVDs risks. Higher dietary diversity may have significant correlation with lower CVDs risks and better health condition. Also it can show that DDS may be useful for investigating correlations between diet quality and some chronic diseases including cardiovascular risks [3, 4, 6].

There have been several definitions for food security. All of them state that food security includes two main parts; physical access (availability of food) and economical access [22, 23]. Regarding the obtained results on the status of food security among HF patients, in current research more than half of the patients (57%) were experiencing degrees of food insecurity. The prevalence of food insecurity in this study is in consistency with prior studies conducted in this region. A systematic review on 31 studies in 2016 indicated that prevalence of food insecurity among Iranian households was about 49% [42]. Moreover, approximately 294.7 million people in south Asia have experienced food insecurity to some extents [43]. Bashir et al. also reported that 23% of the households in Pakistan were found to be food insecure [44]. The situation is getting worse in developing countries as a result of high prices of products and economic crises.

Since food insecurity is associated with several cardio-metabolic problems, including diabetes and obesity [24, 25], interventions aiming to reduce the prevalence of food insecurity can be effective in developing nutritional status of patients with chronic diseases.

Based on findings of this study, subjects with lower educational level and higher waist circumference were more likely to have food insecurity. Additionally, food insecurity was independently associated with gender and household economic condition. Patients with low economic status were more likely to have food insecurity. Prevalence of food insecurity based upon household income/expenditure surveys was 10% according to a meta-analyze experiential/perception-based study in Iran, this systematic review and meta-analysis survey reported rates of mild, moderate and severe food insecurity of 9.3, 5.6 and 3.7%, respectively in 2004 [41]. In the current study, we found that women tended to be more food secure and mild insecure than men; meanwhile, men were more likely to experience severe food insecurity in comparison to women. This finding may be due to the probability that men, as heads of households in this region, pay more attention to other family members’ diet than their own, especially in low-income families struggling with difficult financial access to food.

Chronic diseases are nutrition related and establishing a therapy to prevent and manage chronic diseases is a modification [45–47]. Seligman et al. in their study found a relationship between food insecurity and clinical evidence of hypertension and diabetes [46]. In the study of Vaccaro et al. adults with chronic diseases had greater food insecurity and men had better dietary security state [48], but in present study there was not found any significant correlation between chronic diseases and food insecurity which can be because of the possibility that in our study the overall amount of severe food insecurity was low.

Although the mean of dietary diversity at different levels of food security was higher in patients with complete food security and mild insecurity than patients with moderate to severe insecurity, the difference was not statistically significant. This might be due to the fact that food security questionnaire mostly emphasizes on economic access and the availability of healthy and nutritious food than the diversity of diet.

There are some limitations in this study, such as the cross-sectional design of the study which limits causal relationship between dietary diversity, food security and related factors. Furthermore, although the sample size calculated based on the previous study had a power of 80 and 95% confidence interval, it seems that designing larger prospective cohort studies might be of value in better investigating the determinant factors of dietary diversity and food security.

## Conclusion

Current study showed that patients with HF had low dietary diversity. Also among these patients, those with hypertension, non-smokers and current smokers, those who did not take any supplement, patients with high cholesterol level and lower ejection fraction had lower DDS compared to others. Also, more than half of the heart failure patients were suffering from food insecurity to some extents, of which males and patients with low economic status represents higher levels of food insecurity. Although these findings are preliminary and need further confirmation, it can be mentioned that developing an efficient educational program during hospitalization in order to encourage HF patients to consume a variety of foods within their habitual diet which sufficiently provides essential nutrients can be of high importance. Also, the importance and consequences of high prevalence of food insecurity among HF patients particularly the male subjects call for an urgent action.

## Data Availability

The datasets of the current study are available from the corresponding author on reasonable request.

## Abbreviations

BMI: Body mass index
CVDs: Cardiovascular diseases
DDS: Dietary diversity score
FFQ: Food frequency questionnaire
HF: Heart failure
HFIAS: Household food insecurity access scale
